# Validation-Aware Retrospective EEG Treatment-Response Modelling Using Chaotic Pattern of Prime Numbers Features: Segment-Level Separability and Subject-Wise Generalisation

**DOI:** 10.3390/bioengineering13060659

**Published:** 2026-06-04

**Authors:** Hesam Akbari, Mutlu Mete, Reza Rostami, Reza Kazemi, Muhammad Tariq Sadiq

**Affiliations:** 1 Department of Information Science, University of North Texas, Denton, TX 76203, USA; hesam.akbari@unt.edu (H.A.); mutlu.mete@unt.edu (M.M.); 2 Department of Psychiatry, University of Tehran, Tehran 141556619, Iran; rrostami@ut.ac.ir (R.R.); reza.kazemi@ut.ac.ir (R.K.); 3 School of Computer Science and Electronic Engineering, University of Essex, Colchester CO4 3SQ, UK

**Keywords:** EEG, depression, treatment-response prediction, rTMS, SSRI, CPPN, feature engineering, validation protocol, leave-one-subject-out, subject-wise validation, validation-sensitivity analysis, explainability, feature pattern, research dashboard

## Abstract

This study presents a validation-aware EEG framework based on Chaotic Pattern of Prime Numbers (CPPN) features for depression treatment-response modelling across one SSRI cohort and two rTMS cohorts. CPPN features were evaluated through a seven-protocol validation hierarchy spanning random segment splitting, segment-level cross-validation, nested segment-level cross-validation, leave-N-subjects-out, fixed-feature leave-one-subject-out (LOSO), nested leave-N-subjects-out, and nested LOSO, with normalisation, NCA ranking, feature-count selection where applicable, and model fitting confined to the appropriate training partitions. In the representative K-nearest neighbour (KNN) comparison, segment-level 10-fold CV achieved accuracies of 98.79% for Mumtaz SSRI, 99.32% for small Atieh rTMS, and 99.42% for big Atieh rTMS, demonstrating strong discriminative structure in the CPPN feature space. In the available segment-level KNN comparison, CPPN features with fold-internal NCA-selected feature sets exceeded conventional statistical EEG features by 29.53, 16.33, and 11.45 percentage points across the three cohorts. Subject-wise validation produced lower and more cohort-dependent estimates, with the best fixed-feature LOSO accuracy of 80.00% and the best nested LOSO accuracy of 73.33% in the small Atieh rTMS cohort. These results show that CPPN provides a compact, inspectable and computationally accessible EEG feature representation, while the validation hierarchy gives a transparent account of how performance changes from segment-level separability to held-out-subject evaluation. The main contribution is methodological: this study combines an original CPPN feature representation with explicit validation-depth analysis, leakage-aware feature selection, and interpretable channel/bin inspection. It therefore provides a rigorous basis for future externally validated EEG treatment-response studies without claiming prospective clinical deployment from the present retrospective cohorts.

## 1. Introduction

Mental health disorders have become an increasing global health concern. Among these conditions, depressive disorders represent one of the most frequently diagnosed psychiatric illnesses worldwide [1]. If left untreated, depression can lead to serious consequences, including self-harm and suicide. The World Health Organization currently reports that approximately 332 million people worldwide have depression [2]. Suicide, often a tragic outcome of untreated depression, is currently reported by the World Health Organization as the third leading cause of death among individuals aged 15–29 years worldwide [3].

Selective serotonin reuptake inhibitors (SSRIs) are widely used pharmacological treatments for depression, and repetitive transcranial magnetic stimulation (rTMS) is used in selected patients, particularly when response to standard treatment is inadequate. Both therapy classes have regulatory and clinical use in depression, including U.S. Food and Drug Administration (FDA)-approved indications for specific SSRIs and rTMS protocols [4,5]. However, treatment response varies substantially across individuals because depression is clinically and neurobiologically heterogeneous.

Clinicians often encounter significant challenges when selecting the most appropriate therapy for an individual patient. Treatment selection can involve sequential trial, monitoring, and adjustment, and an ineffective first treatment can prolong symptoms and increase clinical burden. For this reason, pretreatment markers that could support treatment-response research are scientifically important, provided that their evidential scope is not overstated.

Patients are classified into two categories based on their response to treatment: responder (R) and non-responder (NR). Responders are individuals who show noticeable improvement after therapy, while non-responders do not experience significant improvement. Numerous research studies have attempted to predict treatment outcomes using a variety of data modalities, such as genetic markers [6], blood biomarkers [7], neuroimaging techniques [8], and socioeconomic indicators [9].

The electroencephalogram (EEG), one of the previously stated modalities, is a non-invasive, real-time, and extensively accessible neuroimaging technique that records brain activity on the scalp using electrodes. EEG signals have previously been used to forecast how well depression therapies would work.

However, visual categorisation of R and NR categories is challenging because EEG treatment-response signals are noisy, high-dimensional, and subject-specific. Research-grade computational frameworks are therefore needed to study EEG-derived response patterns in a transparent and validation-aware way. In the literature, various studies have employed EEG signals to predict depression-treatment outcomes automatically. A validation-focused comparison of representative studies is provided in Table 1. The table is not intended to dismiss prior work; rather, it clarifies how reported performance should be interpreted according to the validation unit. This distinction is central for EEG machine learning because models may perform strongly when EEG segments or images are randomly partitioned, while still generalising poorly to participants whose recordings were never seen during training.

A second challenge is that EEG treatment-response models are difficult to communicate when their outputs remain as high-dimensional feature vectors, fold-level metrics, or classifier scores. Feature-level outputs are not easily inspectable by non-developers, and this can limit reproducibility, reviewer scrutiny, and translational planning. For this reason, the present framework includes a validation-aware CPPN-EEG research dashboard that links EEG segment previews, CPPN channel/bin feature-map visualisation, and paired SSRI/rTMS cohort-pattern outputs. The dashboard provides an inspection-oriented research software layer for reviewing the engineered feature representation, communicating model behaviour, and supporting future validation workflows with clinical and methodological collaborators.

Taken together, these studies demonstrate the promise of EEG for antidepressant and rTMS treatment-response prediction, but they also show substantial heterogeneity in validation design. In several cases, performance is reported under one principal cross-validation or hold-out setting, and subject-level independence is not always explicit. This matters because EEG segments or images derived from the same individual can share stable subject-specific structure; if such observations are split across training and testing folds, performance may reflect non-independent within-dataset separability rather than generalisation to unseen participants. Recent methodological work has therefore emphasised subject-based validation and nested data partitioning for EEG machine learning [20,21]. The present study addresses this gap by evaluating the CPPN feature representation under seven data-partitioning settings. This design separates protocols that test segment-level separability from subject-wise and nested protocols that provide more conservative estimates of unseen-participant generalisation.

Feature extraction is a critical step in interpreting the intricate patterns present within EEG recordings, as it enhances the relevant signal information while minimising redundant data [22]. In the context of predicting responses to depression treatments, prior research classifies extracted EEG features into three principal categories: time-domain, frequency-domain, and time–frequency features. Frequency-domain methods derive information by evaluating the spectral composition of EEG signals [11,14]. In contrast, time–frequency approaches capture signal characteristics across both temporal and spectral dimensions, often by decomposing EEG into subbands and tracking their variations over time [10,16].

These techniques face several challenges, such as high computational demands, slow processing, and poor performance with non-stationary signals. They also exhibit noise sensitivity, fixed window size limitations, and difficulty detecting localised spikes or selecting an appropriate mother wavelet.

Compared to frequency- and time–frequency-domain methods, direct time-domain feature extraction analyzes the EEG waveform itself through statistical, symbolic, pattern-based or matrix-derived descriptors [11,12,13,23]. These approaches are attractive because they can be computationally simpler than time–frequency image generation or deep transfer-learning pipelines. However, Table 1 also shows that reported performance cannot be judged from accuracy alone. A method that performs well under a segment-level protocol may be useful for identifying discriminative structure in the recordings, but it does not automatically provide evidence of subject-independent prediction. This motivates the use of a feature representation that is both traceable and evaluated under validation protocols with clearly different evidential meanings.

To tackle these issues, we evaluate a distinctive feature extraction technique called the Chaotic Pattern of Prime Numbers (CPPN). This representation uses a fixed prime-indexed sampling pattern to encode local temporal variation in EEG segments.

The CPPN feature extraction approach provides a deterministic local temporal pattern representation of non-stationary EEG segments while maintaining a relatively simple computational structure. Prime-indexed sampling is used as a deterministic, non-uniform time-domain encoding whose evidential value is assessed under explicit validation protocols.

The originality of the proposed framework lies in combining a compact prime-indexed CPPN feature representation with a validation hierarchy, validation-sensitivity analysis, and feature-level inspection layer for EEG treatment-response modelling. Rather than relying only on a single classifier result, this study evaluates how CPPN behaviour changes across segment-level, subject-wise and nested subject-wise protocols. This gives the work broader methodological value: it introduces an inspectable EEG feature-engineering pipeline, tests it across SSRI and rTMS treatment-response cohorts, and links model outputs back to channel/bin-level candidate feature patterns and a visual research dashboard. The intended contribution is a validation-aware methodological framework that exposes how validation design changes the evidence provided by EEG treatment-response models.

The main contributions are: (i) a deterministic CPPN feature-extraction implementation based on 97-sample windows, 25 prime-indexed samples, a 5 × 5 matrix representation, column-variability selection, binary thresholding, and a 32-bin channel-wise histogram; (ii) an available KNN comparison showing that CPPN features with fold-internal NCA-selected feature sets achieve higher segment-level accuracy than conventional statistical EEG features under the same broad classifier and validation family; (iii) a seven-protocol validation hierarchy that separates segment-level separability from subject-wise and nested subject-wise estimates; (iv) a KNN-only validation-sensitivity analysis across segment-level, subject-wise and nested subject-wise settings; and (v) a descriptive inspection and dashboard layer that maps selected CPPN features to EEG channels, local histogram bins and paired SSRI/rTMS response-pattern outputs. Together, these components position the work as a validation-aware EEG feature-engineering and research-software contribution with explicit originality in the CPPN representation, significance in the validation-depth analysis, and rigour in the fold-restricted evaluation design.

The present work is structured as follows: Section 2 describes the datasets, preprocessing, CPPN feature extraction, validation hierarchy, explainability analysis, and research dashboard. Section 3 reports the validation results, baseline comparisons, KNN validation-sensitivity analyses, candidate feature-pattern analysis, and software demonstrator. Section 4 concludes this article.

## 2. Materials and Methods

**Figure 1:**
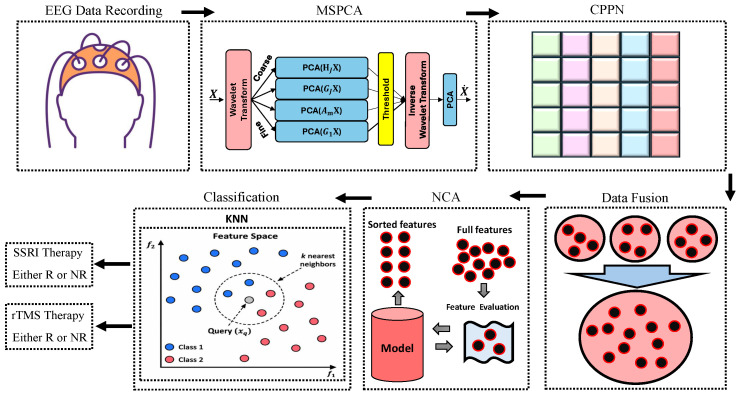
Overall EEG treatment-response classification workflow. The diagram summarises EEG data recording, MSPCA preprocessing, CPPN feature construction, data fusion, NCA-based feature evaluation, and final KNN classification into responder (R) or non-responder (NR) outcomes for SSRI and rTMS therapy cohorts.

This study was designed as a retrospective, validation-aware methodological evaluation of CPPN-based EEG treatment-response prediction from pretreatment EEG. The workflow evaluates signal denoising, non-overlapping 15 s segmentation, CPPN feature extraction, neighbourhood component analysis (NCA)-based feature ranking, classifier performance, segment-level validation, subject-wise validation, nested validation, and validation-aware interpretation of model performance. The overall pipeline is shown in Figure 1.

### 2.1. Study Design and Datasets

Three pretreatment EEG cohorts were analysed separately: one SSRI cohort from the Mumtaz dataset and two rTMS cohorts from Atieh Hospital. The task in all cohorts was binary treatment-response prediction among depressed participants. Class 1 represented responders (R), and Class 2 represented non-responders (NR). Healthy controls were not included because the aim was not depression diagnosis, but prediction of response among participants already diagnosed with depression. Dataset characteristics, segmentation counts, and feature dimensions are summarised in Table 2; cohort-level demographic and clinical characteristics for the Mumtaz SSRI dataset and the large Atieh rTMS dataset are summarised in Table 3 and Table 4, respectively. Demographic data for the small Atieh rTMS cohort (N = 15: 9 responders, 6 non-responders) were not available in sufficient detail for tabulation.

The broader Mumtaz database has been described as containing depressed participants and healthy controls; however, the present treatment-response analysis used only the 30 depressed participants for whom complete pretreatment EEG recordings and responder/non-responder outcome labels were available in the processed response-prediction subset. This subset comprised 12 responders and 18 non-responders. The four additional depressed participants described in the broader Mumtaz database were not part of the complete processed response-prediction subset used here. Healthy controls and records without complete treatment-response modelling information were not included, because the aim was response prediction among depressed participants rather than depression diagnosis. EEG recordings were obtained before SSRI treatment using 20 channels at 256 Hz. Participants were labelled as responders or non-responders according to the treatment-response criterion used in the original dataset study [10,24]. The Atieh Hospital rTMS data were collected before rTMS treatment and consisted of two cohorts. The smaller rTMS cohort included 15 participants, with 9 responders and 6 non-responders. The larger rTMS cohort included 46 participants, with 23 responders and 23 non-responders. Both rTMS cohorts used 19 EEG channels sampled at 500 Hz, and response labels were determined from pre-treatment and post-treatment clinical assessment. The 10–20 electrode system and channel groupings used in the datasets followed the original acquisition protocols.

### 2.2. Preprocessing and Segmentation

All datasets were processed at their native sampling rates: 256 Hz for the Mumtaz SSRI dataset and 500 Hz for the Atieh Hospital rTMS datasets. No resampling was applied. No additional re-referencing was performed in the computational pipeline; the original referencing scheme of each dataset was retained. Filtering was performed in EEGLAB [25] to reduce low-frequency drift, high-frequency noise, and power-line interference. A 0.5 Hz high-pass finite impulse response (FIR) filter, a 45 Hz low-pass FIR filter, and a 47–53 Hz band-stop filter were applied. The subsequent analysis was therefore restricted to the conventional EEG range retained by this filtering stage; no additional frequency-band selection or band-specific feature extraction was applied before CPPN feature construction. Gross non-neural contamination was addressed through the available preprocessed/denoised EEG records and MSPCA denoising rather than through a separate manual independent-component rejection step, ensuring that the same automated preprocessing route was applied consistently to all cohorts. No manual ICA-based artefact rejection or visual trial rejection was added in the present pipeline; this choice preserved a consistent automated preprocessing pathway across cohorts but is also a methodological boundary for future work.

**Figure 2:**
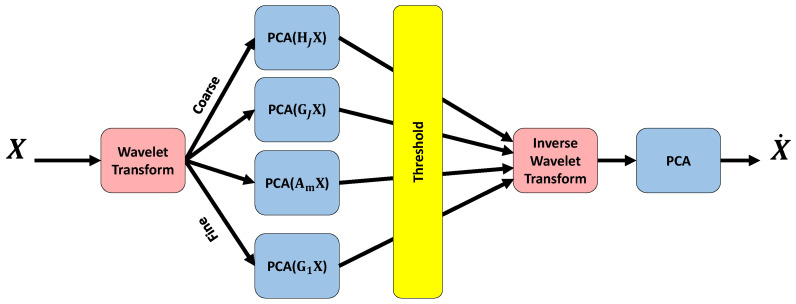
MSPCA denoising workflow used for multichannel EEG preprocessing.

After filtering, multiscale principal component analysis (MSPCA) was used for additional denoising. MSPCA combines multiscale wavelet decomposition with PCA-based coefficient selection and reconstruction, and is commonly used to suppress noise in non-stationary multichannel signals [26,27]. In this implementation, MSPCA was applied to the complete multichannel record before segmentation. The wavelet-decomposed coefficient matrices were subjected to PCA, noise-dominated components were rejected according to the Kaiser criterion, and the denoised signal was reconstructed by inverse wavelet transform followed by PCA reconstruction. This made the denoising step deterministic and identical across cohorts: the same filter range, no resampling, no extra re-referencing, and the same coefficient-retention rule were used before non-overlapping 15 s segmentation. The MSPCA workflow is shown in Figure 2.

Each EEG record was divided into non-overlapping 15 s segments. This produced 579 segments in the Mumtaz SSRI cohort, 294 segments in the small Atieh rTMS cohort, and 856 segments in the big Atieh rTMS cohort. At the segment level, the Mumtaz data contained 230 responder-labelled and 349 non-responder-labelled segments; the small Atieh data contained 182 responder-labelled and 112 non-responder-labelled segments; and the big Atieh data contained 437 responder-labelled and 419 non-responder-labelled segments. A Mumtaz segment therefore had dimensionality 20×3840 samples, whereas an Atieh segment had dimensionality 19×7500 samples. In the notation below, $N_{s}$ denotes the number of samples in a single-channel segment: $N_{s}=3840$ for Mumtaz and $N_{s}=7500$ for the Atieh cohorts.

### 2.3. CPPN/Prime-Pattern Feature Extraction

The Chaotic Pattern of Prime Numbers (CPPN) representation is the central feature-engineering contribution of this study. CPPN provides a deterministic, non-uniform, prime-indexed time-domain encoding of local EEG morphology and was applied separately to each EEG channel within each 15 s segment. Its rationale is signal-processing rather than purely numerological: prime-indexed sampling creates a fixed irregular temporal mask inside each local window, reducing the dependence on uniformly spaced samples while preserving short-range morphology. The subsequent column-variability operation converts this non-uniform local sampling into a compact binary pattern and a 32-bin histogram. CPPN therefore differs from entropy features, which summarise signal unpredictability, and from wavelet or time–frequency features, which summarise energy across scales or frequencies. CPPN instead encodes recurrent local temporal contrast patterns in a channel-resolved, fixed-length representation. This provides a fixed-dimensional engineered representation while retaining a direct mapping back to EEG channel and CPPN-bin identity. Unlike a convolutional neural network (CNN), where the filter bank and feature extractor are learned and architecture-dependent, CPPN uses a deterministic 32-bin histogram per channel before optional feature ranking and classification. This distinction does not imply that CPPN is universally simpler or superior to CNN-based models; rather, it defines a different feature-engineering route whose value is assessed empirically under the validation hierarchy used in this study.

For one EEG channel x[n] with $N_{s}$ samples, CPPN uses overlapping local blocks of 97 samples:

(1) $$b_{t}\left(j\right)=x\left[t+j-1\right],\;j=1,2,\ldots ,97,\;t=1,2,\ldots ,N_{s}-96.$$

The first 25 prime indices,

(2) PN={2,3,5,7,11,13,17,19,23,29,31,37,41,43,47,53,59,61,67,71,73,79,83,89,97},

were used to sample each 97-sample local block. The 25 sampled values were reshaped into a 5×5 matrix. The standard deviation of each column was computed, the two columns with the highest variability were selected, and their difference produced a five-element vector $S_{t}$. A local threshold was defined from the standard deviation of the 97-sample block, and each element of $S_{t}$ was binarised relative to this threshold. The five binary values were converted to a decimal CPPN code:

(3) $$CPPN\left(t\right)=\sum_{k=1}^{5}f\left(S_{t}\left(k\right),\gamma_{t}\right)2^{k-1},\;f\left(\alpha ,\beta \right)=\left\{\begin{array}{cc} 1, & \alpha \geq \beta ,\\ 0, & \alpha <\beta . \end{array}\right.$$

**Figure 3:**
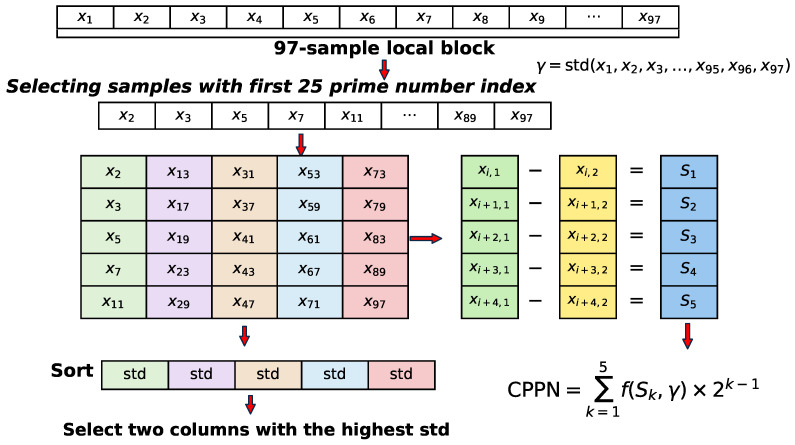
CPPN feature extraction schematic. A 97-sample local block is sampled at the first 25 prime-number indices, reshaped into a 5×5 matrix, reduced through column-variability selection, and encoded as a 32-bin channel-wise histogram.

This produced integer pattern values in the range of 0–31. A 32-bin histogram of these values was then computed for each channel. The CPPN feature-construction procedure is illustrated in Figure 3.

### 2.4. Data Fusion and Statistical-Feature Baseline

**Figure 4:**
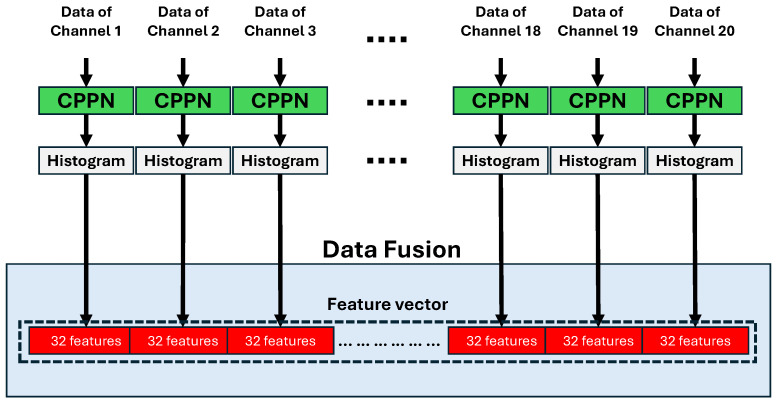
Channel-wise CPPN feature fusion. A 32-bin CPPN histogram is extracted from each EEG channel and concatenated to form the final segment-level feature vector. The figure illustrates the 20-channel Mumtaz SSRI structure; the Atieh rTMS datasets use 19 channels and follow the same procedure, producing 608 concatenated features.

Each EEG channel generated a 32-bin CPPN histogram. Channel-wise data fusion was performed by concatenating the histograms across all EEG channels:

(4) $$F=[H_{Ch1},H_{Ch2},\ldots ,H_{Chn}],$$

where $H_{Chi}$ denotes the CPPN histogram extracted from channel *i*, and *n* is the number of channels. This produced 640 CPPN features for the 20-channel Mumtaz SSRI dataset and 608 CPPN features for each 19-channel Atieh rTMS dataset. Thus, each channel contributed exactly 32 deterministic CPPN histogram features. In contrast to a one-dimensional CNN that would learn convolutional filters and feature maps from data, CPPN produces a fixed engineered 32-bin channel descriptor before supervised model fitting. This distinction is important for complexity interpretation: CPPN reduces each 15 s channel segment to 32 non-trainable histogram features, whereas CNN-based alternatives require fold-internal training and tuning of the convolutional architecture. The channel-wise concatenation procedure is shown in Figure 4.

A conventional statistical-feature baseline was also evaluated to compare CPPN with simpler handcrafted descriptors. Seven descriptors were extracted from each channel: mean, standard deviation, variance, skewness, kurtosis, minimum, and maximum. This yielded 140 statistical features for the 20-channel Mumtaz SSRI dataset and 133 statistical features for each 19-channel Atieh rTMS dataset. The statistical-feature baseline provided a transparent comparator for assessing the added value of the CPPN representation.

For the available conventional-feature comparison, statistical EEG features and CPPN-derived features were evaluated using the same downstream classifier setting under segment-level 10-fold cross-validation with training-fold-only normalisation. The statistical feature sets contained 579×140 features for the Mumtaz SSRI dataset, 294×133 features for the small Atieh rTMS dataset, and 856×133 features for the big Atieh rTMS dataset. The CPPN side used the full CPPN feature matrix as the source representation but applied NCA feature ranking inside the training folds before downstream classification, using the fixed feature counts reported in Section 3.6. No global supervised feature selection was fitted on the full dataset before cross-validation. This setting provides a controlled within-dataset comparison of feature separability under the same broad classifier and validation family, but the CPPN side should be read as fold-internal NCA-selected CPPN with the downstream classifier rather than an unselected full-feature CPPN comparison.

### 2.5. Feature Ranking and Feature Selection

Neighbourhood Component Analysis (NCA) was used to rank CPPN features according to their supervised discriminative contribution [28]. CPPN initially produced 640 features for Mumtaz SSRI and 608 features for each Atieh rTMS cohort. To avoid data leakage, all z-score normalisation parameters, NCA feature weights, selected feature counts, and classifier parameters were estimated strictly inside the training portion of each validation split. Held-out segments or held-out subjects were never used for normalisation, NCA fitting, feature-count selection, hyperparameter choice, or classifier training. In nested protocols, feature-count selection was confined to the inner loop and the outer test fold remained untouched until the final evaluation. This fold-internal procedure is essential because supervised feature selection performed before cross-validation can lead to optimistic performance estimates [29,30,31]. Signal filtering and MSPCA denoising were applied before segmentation as fixed unsupervised preprocessing steps; fold-wise leakage control in the classifier comparisons refers to scaling, supervised feature selection where applicable, and classifier fitting.

For fixed-feature validation protocols, the feature counts were pre-specified before testing: 243 features for the Mumtaz SSRI dataset, 587 features for the small Atieh rTMS dataset, and 377 features for the big Atieh rTMS dataset. For nested validation protocols, feature-count selection was performed only inside the inner training/validation loop. The candidate feature counts were 100, 200, 243, and 300 for Mumtaz SSRI; 56, 95, 300, 377, and 587 for small Atieh rTMS; and 95, 300, 377, and 587 for big Atieh rTMS. Outer test subjects in nested subject-wise protocols remained untouched until final evaluation.

### 2.6. Classifiers

The main classifier analysis evaluates CPPN-derived feature representations using K-nearest neighbour (KNN) under the validation hierarchy described below. The reported KNN analyses include segment-level, subject-wise, and nested validation settings with fold-internal normalisation and supervised feature selection where applicable. Non-KNN classifier families are not used as primary results in the present manuscript because the current validation hierarchy is reported with KNN outputs only.

Two KNN settings were used. First, CPPN feature vectors were evaluated under segment-level 10-fold CV with training-fold-only normalisation and model fitting to test controlled within-dataset segment separability. Second, subject-wise KNN analyses were performed under fixed-feature LOSO and nested LOSO. In the subject-wise analyses, all subject separation rules were preserved, and subject-level decisions were obtained by majority vote across the held-out subject’s segment predictions. For nested LOSO, feature-count/model selection was performed only within the training subjects of each outer fold. These analyses are interpreted as validation-sensitivity checks that show how KNN performance changes from segment-level separability to held-out-subject evaluation.

### 2.7. Validation Protocol Hierarchy

**Figure 5:**
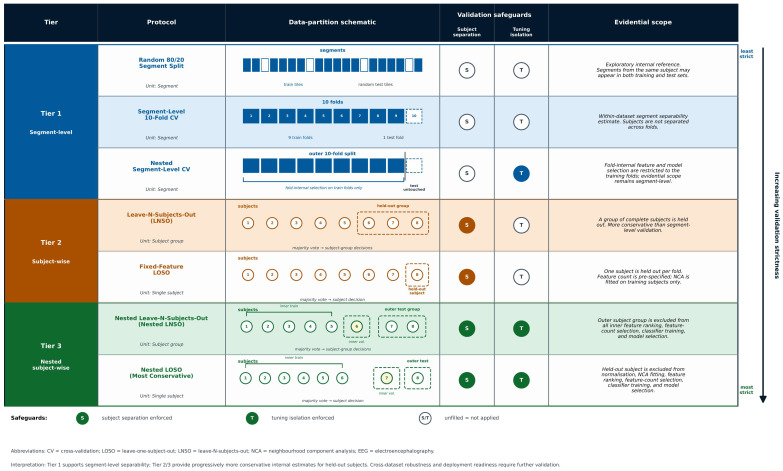
Validation hierarchy used to evaluate the CPPN framework. Tier 1 contains segment-level protocols, Tier 2 contains subject-wise protocols, and Tier 3 contains nested subject-wise protocols with explicit separation between inner validation and outer held-out testing. Filled safeguard markers indicate subject separation and tuning isolation. The hierarchy supports a transparent reading of how CPPN performance changes from segment-level feature separability to increasingly conservative held-out-subject evidence.

The evaluation was organised around the validation hierarchy shown in Figure 5. The hierarchy separates seven data-partitioning protocols into three tiers so that each accuracy value is interpreted according to the validation question it answers. Tier 1 evaluates segment-level feature separability, Tier 2 evaluates held-out subjects or subject groups, and Tier 3 adds nested model-selection control inside the subject-wise setting. This structure provides a rigorous and transparent way to show how CPPN performance changes as the evaluation moves from segment discrimination to more conservative held-out-participant testing.

Tier 1 contains Random 80/20 Segment Split, Segment-Level 10-Fold CV, and Nested Segment-Level CV. These protocols use EEG segments as the split unit and are useful for assessing whether the engineered CPPN representation forms a discriminative segment-level feature space. The nested segment-level protocol additionally restricts feature/model selection to the training folds, which strengthens the internal segment-level estimate while retaining the segment as the validation unit.

Tier 2 changes the validation unit from segments to complete participants. Leave-n-subjects-out holds out a subject group, whereas Fixed-Feature LOSO holds out one complete subject per fold after the feature count has been pre-specified. In both protocols, all segments belonging to the held-out subject or subject group are excluded from training and are aggregated by majority voting to form a subject-level or subject-group decision.

Tier 3 combines subject separation with tuning isolation. Nested Leave-N-Subjects-Out holds out a subject group in the outer loop and performs feature/model selection only inside the remaining outer-training subjects. Nested LOSO holds out one subject as the final outer-test subject and uses only the training subjects for normalisation, NCA fitting, feature ranking, feature-count selection, classifier training, and model selection. This tier is the strictest internal validation setting used in the manuscript and is particularly important whenever supervised ranking or parameter tuning is part of the pipeline.

The safeguard columns in Figure 5 distinguish subject separation from tuning isolation. Subject separation indicates that complete participants are separated between training and testing. Tuning isolation indicates that feature/model selection is performed inside the training portion of the validation loop, without access to the final held-out segments or subjects. Together, Figure 5 and Table 5 define the evidential scope used throughout the Results section. The seven protocols were used as a validation hierarchy rather than as equivalent estimates of clinical performance; segment-level protocols test within-dataset separability, whereas subject-wise and nested subject-wise protocols provide progressively stricter internal held-out-subject evidence.

For the CPPN-only validation-sensitivity analysis, Tier 2 fixed-feature LOSO used pre-specified feature counts and common KNN settings. Tier 3 nested LOSO used the same outer held-out-subject structure, but classifier parameters and feature-count selection were selected using only the outer-training subjects before final testing on the held-out subject. The subject-wise analysis reported in the Results focuses on KNN-only outputs, avoiding any interpretation as a multi-classifier comparison while preserving the validation hierarchy defined above.

Across all subject-wise protocols, “window-level prediction” refers to prediction for an individual 15 s EEG segment/window. Subject-majority prediction refers to the final subject-level decision obtained by majority voting across all segment/window predictions belonging to the held-out subject. This distinction allows this study to report both segment-level behaviour and subject-level treatment-response prediction.

### 2.8. Performance Metrics and Statistical Analysis

Classification performance was assessed using accuracy, sensitivity, specificity, precision, recall, F1-score, balanced accuracy, and confusion-matrix counts. A responder was treated as the positive class. True positives (TP) were responder segments or subjects correctly classified as responders; true negatives (TN) were non-responder cases correctly classified as non-responders; false positives (FP) were non-responders incorrectly classified as responders; and false negatives (FN) were responders incorrectly classified as non-responders.

The main proportion-based metrics were computed as follows:

(5) $$Accuracy=\frac{TP+TN}{TP+TN+FP+FN},\;Sensitivity=\frac{TP}{TP+FN},\;Specificity=\frac{TN}{TN+FP}.$$

Precision, recall, F1-score, and balanced accuracy were computed from the same confusion counts when reported. Wilson 95% confidence intervals were used for accuracy, sensitivity, and specificity. Repeated random-split results were summarised using mean, standard deviation, median, interquartile range (IQR), minimum, and maximum. Subject-wise protocols reported both pooled segment-level metrics and subject-majority metrics. Subject-majority predictions were obtained by majority vote across all segment predictions from the held-out subject.

### 2.9. Explainability and Candidate EEG-Derived Feature-Pattern Analysis

Selected CPPN features were mapped back to their original EEG channels and CPPN histogram bins. Because the CPPN feature vector was constructed by concatenating channel-wise 32-bin histograms, each selected feature retained a direct channel/bin identity. This mapping allowed the CPPN representation to be inspected as an organised EEG feature space rather than as an opaque vector of selected predictors. Channel contribution counts and channel × bin contribution maps were therefore computed to summarise which EEG channels and local CPPN histogram bins contributed most frequently to the selected feature sets. These maps were descriptive feature-level outputs of the feature-selection process and should not be interpreted as SHAP or LIME explanations of individual predictions [32,33].

An exploratory candidate EEG-derived feature-pattern analysis was additionally performed at the subject level, because treatment response is a subject-level outcome. Existing CPPN feature matrices were used; raw EEG preprocessing and CPPN feature extraction were not rerun for this analysis. Segment-level CPPN features were aggregated within each participant, and responder/non-responder association was assessed using effect-size estimates, fold-wise training data, and false-discovery-rate (FDR) correction. Held-out subject candidate feature-pattern scores and LOSO channel/bin selection-frequency maps were then used to describe candidate explanatory patterns. Where no FDR-selected feature survived within a LOSO training fold, fallback-ranked candidate features were used to visualise exploratory channel/bin recurrence. The aim was to provide a transparent, inspectable route from CPPN classification features back to EEG channel/bin structures and to create candidate feature patterns that can be prioritised for clinician review, test–retest analysis, and future prospective validation.

### 2.10. Validation-Aware Research Dashboard and Software Demonstrator

A MATLAB Version 2025-based CPPN-EEG research dashboard was developed as a software demonstrator for structured feature inspection and communication of the model workflow. The dashboard uses the CPPN feature matrices generated from the three cohorts and displays four linked elements: an EEG segment preview, a CPPN channel/bin feature-map visualisation, an SSRI cohort-pattern score, and an rTMS cohort-pattern score. Because the CPPN representation concatenates 32 histogram bins per EEG channel, the software can reshape the segment-level CPPN feature vector into a channel × bin map, allowing the representation to be inspected as an organised feature image rather than only as a numerical vector.

The dashboard was designed to visualise four paired exploratory response-pattern scenarios: SSRI responder-like/rTMS responder-like, SSRI non-responder-like/rTMS non-responder-like, SSRI responder-like/rTMS non-responder-like, and SSRI non-responder-like/rTMS responder-like. The displayed cohort-pattern scores communicate model behaviour and feature-space structure across the retrospective cohorts. The software layer supports transparent inspection of the CPPN representation, structured reporting of model outputs, and communication with researchers, clinicians, collaborators, and future validation teams.

## 3. Results and Discussion

The results are reported according to the validation hierarchy defined in Section 2.7. This organisation is necessary because the seven protocols do not estimate the same quantity. Tier 1 protocols evaluate within-dataset segment-level separability; Tier 2 protocols evaluate transfer to held-out subjects or subject groups without full inner model-selection isolation; and Tier 3 protocols provide nested subject-wise estimates in which model or feature-count selection is separated from final testing. Table 6 first provides the complete seven-protocol KNN validation summary. The following sections then focus on the representative segment-level 10-fold CV, fixed-feature LOSO, and nested LOSO comparison, followed by statistical-feature baselines, KNN-only subject-wise validation checks, a compact nested segment-level feature-selection control analysis, explainability, and comparison with previous EEG treatment-response studies.

### 3.1. Representative KNN Comparison Across Segment-Level, Fixed-Feature LOSO and Nested LOSO Validation

**Figure 6:**
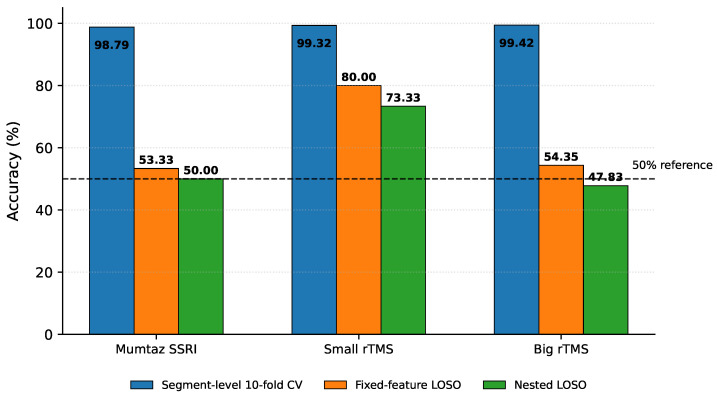
CPPN + KNN performance across the validation hierarchy. Segment-level 10-fold validation shows strong CPPN separability in all three cohorts, while fixed-feature LOSO and nested LOSO provide held-out-subject estimates. The figure highlights the segment-to-subject validation transition and the importance of nested model-selection control.

After the seven-protocol validation summary, Table 7 and Figure 6 focus on three representative KNN protocols: segment-level 10-fold CV, fixed-feature LOSO, and nested LOSO. The segment-level and subject-wise analyses estimate different quantities. The segment-level 10-fold experiment tests whether CPPN features separate EEG segments within the available recordings. In that experiment, no global NCA was used, no supervised feature selection was fitted on the full dataset, and normalisation was fitted only on the training folds. Because the split unit is the EEG segment, this protocol is best read as a controlled Tier 1 estimate of within-dataset feature separability, with fold-wise normalisation and model fitting restricted to the training folds.

The Tier 2 and Tier 3 KNN results are stricter because the test unit is a complete held-out subject. Tier 2 uses fixed-feature LOSO with common KNN settings, while Tier 3 uses nested LOSO, where classifier-parameter selection is confined to training subjects within each outer fold. The contrast between the high segment-level KNN accuracies and the lower LOSO accuracies is the central validation message of the present analysis.

Segment-level KNN performance was high in all three cohorts, reaching 98.79% in Mumtaz SSRI, 99.32% in small Atieh rTMS, and 99.42% in big Atieh rTMS. In contrast, subject-wise KNN performance was lower and more cohort-dependent. Under fixed-feature LOSO, KNN achieved 53.33% in Mumtaz SSRI, 80.00% in small Atieh rTMS, and 54.35% in big Atieh rTMS. Under nested LOSO, the corresponding KNN accuracies were 50.00%, 73.33%, and 47.83%. These results show that high segment-level separability does not automatically imply high unseen-subject generalisation.

**Figure 7:**
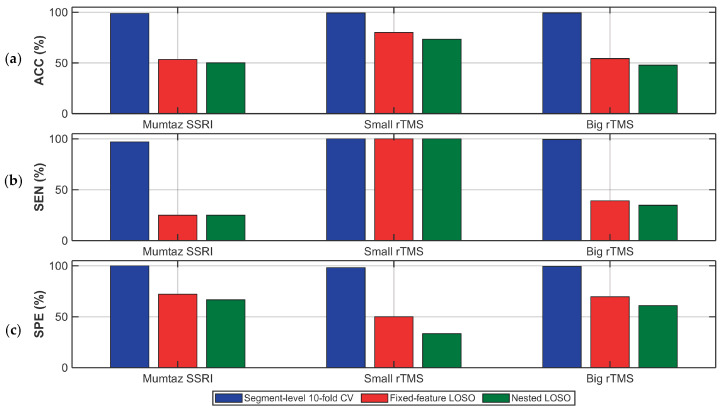
CPPN + KNN metric profiles across the validation hierarchy: (**a**) accuracy, (**b**) sensitivity, and (**c**) specificity. Segment-level 10-fold CV summarises within-dataset segment separability, whereas fixed-feature LOSO and nested LOSO summarise held-out-subject performance. The profile shows that the three metrics vary across cohorts and validation protocols.

Figure 7 further shows that the KNN-based CPPN performance profile differs across accuracy, sensitivity, and specificity. This is important because a single accuracy value can hide asymmetric sensitivity or specificity behaviour, especially under subject-wise validation.

### 3.2. Tier 1–3 Interpretation

The KNN-based comparison changes the interpretation of the validation hierarchy. Segment-level 10-fold CV shows that the CPPN representation is strongly separable at the segment level. Fixed-feature LOSO shows that complete held-out subjects are harder to classify than held-out segments. Nested LOSO shows the effect of adding inner subject-wise feature-count/model selection within the training subjects only. The drop from segment-level KNN accuracies above 98% to nested LOSO accuracies of 47.83–73.33% demonstrates that the validation levels answer different scientific questions. The present analysis therefore treats KNN segment-level results as evidence of within-dataset separability and LOSO/nested LOSO results as the relevant internal evidence for held-out-subject performance.

### 3.3. KNN-Only Subject-Wise Validation Check

**Figure 8:**
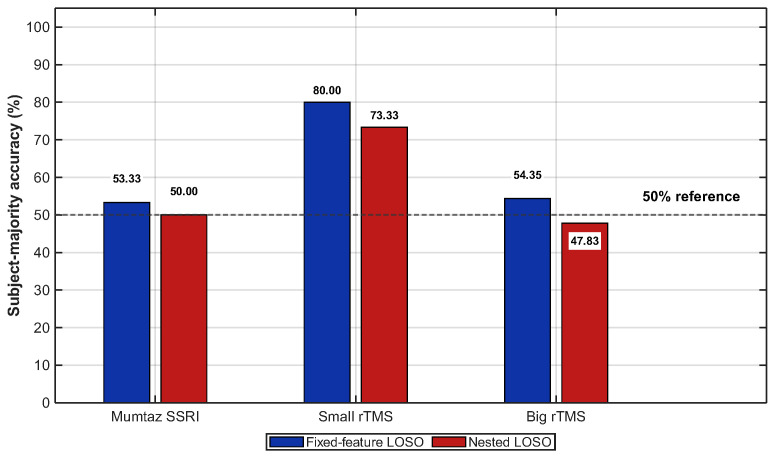
KNN-only subject-wise validation comparison under fixed-feature LOSO and nested LOSO. Bars show subject-majority accuracy for the three EEG treatment-response cohorts. The figure is not a multi-classifier heatmap; it reports only the KNN subject-wise validation outputs.

Table 8, Table 9 and Table 10, together with Figure 8, present the KNN-only subject-wise validation check under fixed-feature LOSO and nested LOSO. This analysis evaluates the CPPN + KNN representation under fixed-feature LOSO and nested LOSO subject-wise validation. It should not be read as a multi-classifier comparison. Instead, it tests how the same CPPN + KNN representation behaves when the validation design changes from fixed-feature held-out-subject evaluation to nested held-out-subject evaluation.

The Mumtaz SSRI cohort achieved 53.33% subject-majority accuracy under fixed-feature LOSO and 50.00% under nested LOSO. The small Atieh rTMS cohort produced the strongest subject-wise KNN performance, with 80.00% under fixed-feature LOSO and 73.33% under nested LOSO. The larger Atieh rTMS cohort showed more modest subject-wise performance, with 54.35% under fixed-feature LOSO and 47.83% under nested LOSO. These findings reinforce the main validation message: CPPN + KNN gives strong segment-level separability, but held-out-subject performance is cohort-dependent and must be interpreted under the appropriate validation protocol.

### 3.4. Matched KNN Statistical-Feature Baseline Comparison

Table 11 presents an available KNN segment-level comparison between conventional statistical EEG features and CPPN features under segment-level 10-fold cross-validation with training-fold-only preprocessing. Statistical EEG features achieved accuracies of 69.26%, 82.99%, and 87.97% for the Mumtaz SSRI, small Atieh rTMS, and big Atieh rTMS cohorts, respectively. Using segment-level 10-fold CPPN + KNN values in which NCA ranking was fitted inside the training folds and the fixed feature counts were 243, 587, and 377, the CPPN-derived representation achieved higher accuracies of 98.79%, 99.32%, and 99.42%, corresponding to improvements of 29.53, 16.33, and 11.45 percentage points. These findings support CPPN as a stronger segment-level feature representation than the conventional statistical feature set in this available KNN comparison. Because this protocol is segment-level, the results are interpreted as within-dataset segment separability rather than subject-independent generalisation.

**Figure 9:**
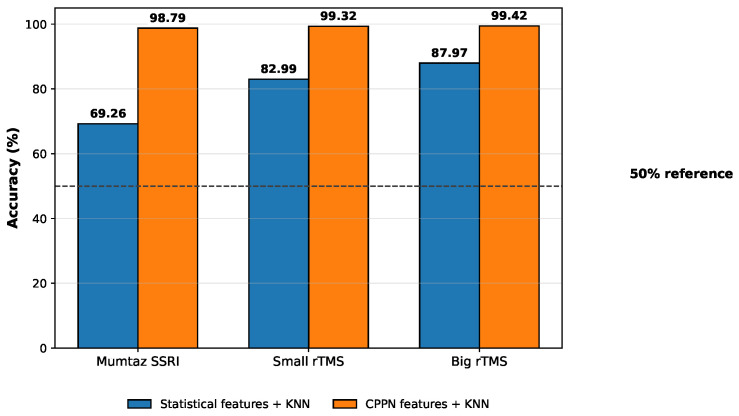
Available KNN comparison between conventional statistical EEG features and CPPN features under segment-level 10-fold cross-validation with training-fold-only preprocessing. CPPN values use fold-internal NCA-selected feature sets, whereas the statistical-feature baseline uses conventional statistical feature matrices. The comparison supports the discriminative value of the CPPN representation under a segment-level benchmark, but it is not an unselected full-feature CPPN comparison.

Figure 9 visualises the same available KNN accuracy comparison using the segment-level CPPN values. The consistent accuracy gain across all three cohorts indicates that CPPN features capture discriminative segment-level structure beyond the conventional statistical feature set under this segment-level KNN comparison with fold-internal NCA selection on the CPPN side.

### 3.5. Seven-Protocol KNN Validation Heatmap

**Figure 10:**
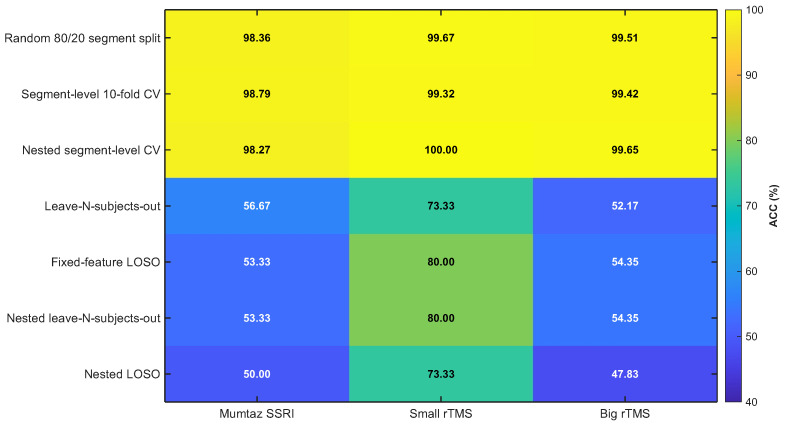
KNN-only seven-protocol validation heatmap. Rows show the seven validation protocols and columns show the three EEG treatment-response cohorts. Cell values are the main reported accuracy for each protocol: repeat-mean segment accuracy for random 80/20 splitting, pooled segment accuracy for segment-level protocols, and subject-majority accuracy for subject-wise protocols.

Figure 10 visualises the seven-protocol KNN validation hierarchy across the three cohorts. The heatmap complements Table 6 by showing how apparent performance changes as the validation design becomes stricter. Segment-level protocols remain high across cohorts, whereas subject-wise and nested subject-wise protocols are lower and more cohort-dependent. This figure is a KNN-only validation heatmap; it is not a Decision Tree, Random Forest, SVM, or LDA classifier-comparison figure.

### 3.6. KNN-Based NCA Feature-Selection Control

To assess whether high segment-level performance depended on non-nested feature-count selection, we compared segment-level 10-fold CV with nested segment-level CV. The compact comparison in Table 12 shows that CPPN + KNN retained high segment-level separability after normalisation, NCA feature ranking, feature-count selection, and classifier training were moved inside the outer-training folds. These results remain segment-level estimates and should not be interpreted as subject-independent generalisation.

### 3.7. Explainability and Candidate EEG-Derived Feature-Pattern Interpretation

Beyond classification performance, the CPPN representation provides an important explainability advantage because each selected CPPN feature can be traced back to a specific EEG channel and CPPN histogram bin. This allows model behaviour to be inspected at a feature level rather than being reported only as a single classification accuracy value. In this study, the explainability analysis was used to examine how CPPN-derived channel/bin patterns contributed to responder and non-responder separability across the SSRI, rTMS-small, and rTMS-big cohorts.

**Figure 11:**
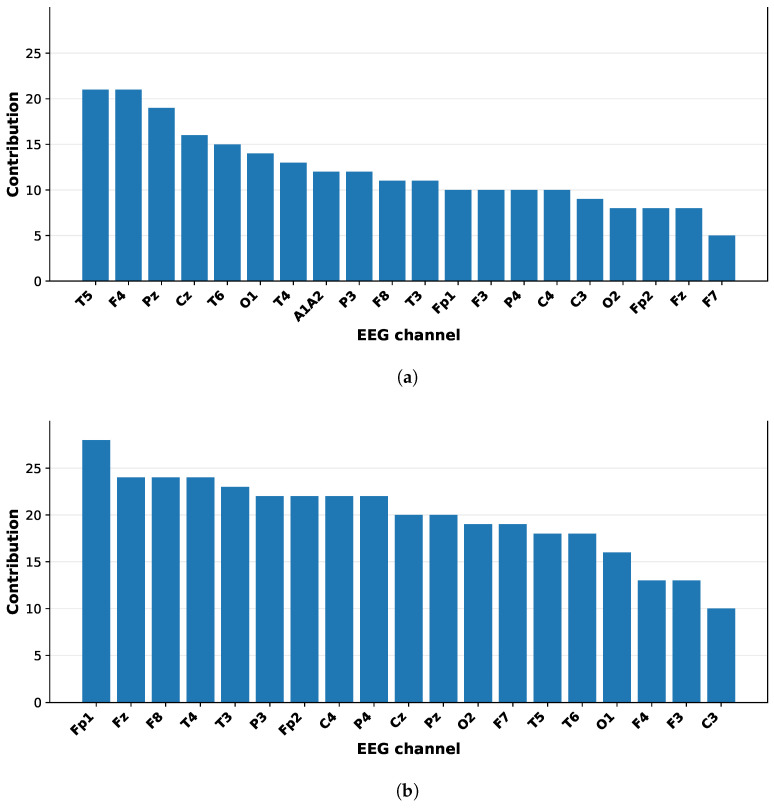
Channel-wise distribution of selected CPPN features for the SSRI and rTMS analyses. (**a**) Channel contribution for the Mumtaz SSRI cohort; the highest contributions were observed for T5, F4, Pz, Cz, and T6. (**b**) Channel contribution for the rTMS cohort analyses (small and large Atieh rTMS datasets); the highest contributions were observed for Fp1, Fz, F8, T4, and T3. Contribution is defined as the number of NCA-selected CPPN features assigned to each EEG channel.

Figure 11 summarises the channel-wise distribution of selected CPPN features. The selected channels were not uniformly distributed across the feature space. Instead, the contribution counts formed concentrated channel structures, supporting the interpretation that CPPN organises EEG segment information into inspectable local pattern groups. These plots are descriptive feature-level outputs of the CPPN–NCA pipeline and should not be interpreted as SHAP, LIME, grouped permutation importance, or biomarker validation.

**Figure 12:**
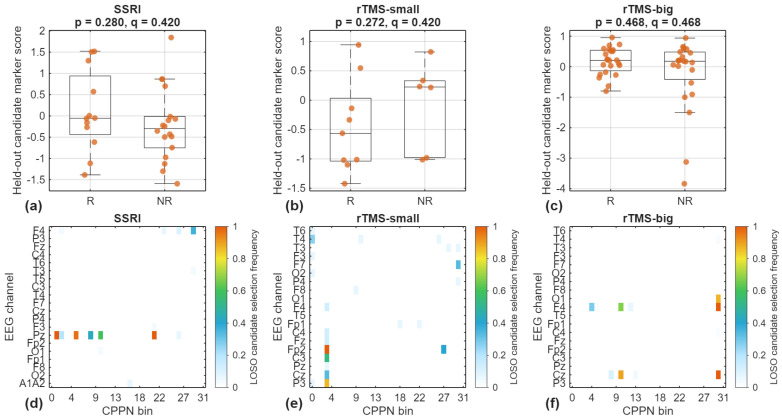
Exploratorycandidate EEG-derived feature-pattern analysis. (**a**–**c**) Subject-level held-out candidate feature-pattern score distributions for the SSRI, rTMS-small, and rTMS-big cohorts, respectively, with responder and non-responder groups shown separately; no FDR-corrected subject-level significance was reached in any cohort. (**d**–**f**) Fallback-ranked exploratory LOSO candidate channel/bin selection-frequency maps for the SSRI, rTMS-small, and rTMS-big cohorts, respectively. These maps show recurrent channel/bin patterns selected within leave-one-subject-out training folds when no FDR-selected features survived. They are hypothesis-generating channel/bin maps that provide a structured basis for clinician review and independent prospective validation.

Figure 12 extends this interpretation to subject-level candidate feature-pattern scores and channel/bin-resolved LOSO recurrence maps. Panels (a–c) show the held-out candidate feature-pattern score distributions for the SSRI, rTMS-small, and rTMS-big cohorts. The responder and non-responder score distributions overlapped in all three cohorts, and no cohort reached FDR-corrected subject-level significance (SSRI: p=0.280, q=0.420; rTMS-small: p=0.272, q=0.420; rTMS-big: p=0.468, q=0.468). The value of these panels is therefore explanatory rather than confirmatory: they show how the segment-level CPPN representation can be summarised into inspectable subject-level candidate scores while preserving the link to the underlying EEG-derived features.

Panels (d–f) of Figure 12 show fallback-ranked exploratory LOSO candidate channel/bin selection-frequency maps. These maps identify which EEG channels and CPPN bins were repeatedly selected across leave-one-subject-out training folds. The selected regions were sparse and cohort-specific rather than uniformly distributed across all channels and bins. This supports the view that CPPN is not simply increasing feature dimensionality, but is creating an inspectable representation in which treatment-response-related structure can be examined at the channel and pattern-bin level. Because these maps were generated using fallback-ranked candidate features when no FDR-selected features survived, they are interpreted as hypothesis-generating feature-level patterns rather than FDR-significant biomarker maps.

Taken together, these findings strengthen the methodological contribution of the CPPN framework. CPPN provides not only a discriminative feature representation for EEG treatment-response classification, but also an inspectable route from classification output back to specific EEG channel and CPPN-bin candidate patterns. This makes the approach more transparent than a purely opaque feature vector and provides a meaningful explanatory layer for understanding how CPPN-derived features contribute to treatment-response separability across SSRI and rTMS datasets. Table 13 provides a structured interpretability summary across cohorts.

These patterns provide an interpretable bridge between CPPN feature selection and cohort-level response structure. The candidate-marker values and channel/bin maps are retained as exploratory outputs from the curated candidate-marker analysis. They are descriptive and require independent validation before clinical interpretation.

### 3.8. CPPN-EEG Research Dashboard

**Figure 13:**
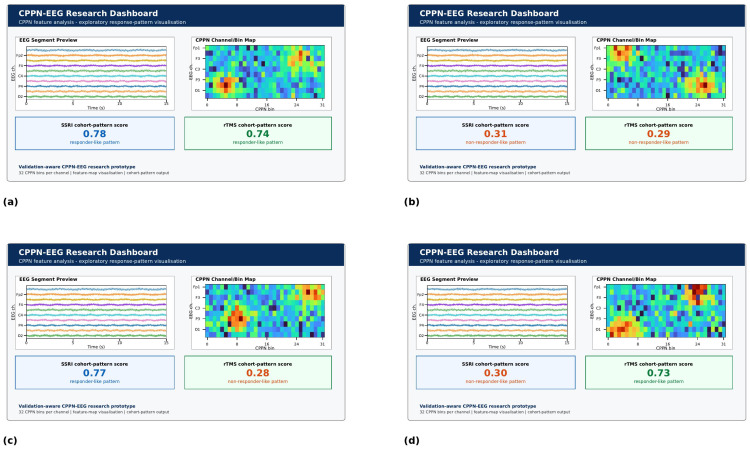
Validation-aware CPPN-EEG research dashboard. The enlarged four-panel dashboard provides EEG segment preview, CPPN channel/bin feature-map visualisation, and exploratory SSRI/rTMS cohort-pattern outputs. (**a**) SSRI and rTMS responder-like pattern outputs. (**b**) SSRI and rTMS non-responder-like pattern outputs. (**c**) SSRI responder-like and rTMS non-responderlike pattern outputs. (**d**) SSRI non-responder-like and rTMS responder-like pattern outputs. The dashboard is intended for transparent inspection, communication of model behaviour, and planning of future clinician-informed validation; it is not a clinical decision-support system in its present form.

The implemented framework includes a validation-aware CPPN-EEG research dashboard that provides an inspectable software layer for the feature-engineering and response-pattern workflow. The dashboard links EEG segment preview, CPPN channel/bin feature-map visualisation, and paired SSRI/rTMS cohort-pattern outputs in a single interface. Figure 13 illustrates the four paired output scenarios generated by the demonstrator. Compared with a static pipeline view, the dashboard makes the CPPN feature space easier to inspect because the same interface connects the EEG segment display, channel/bin-level CPPN representation, and cohort-pattern score outputs. This supports structured visual inspection of the feature representation and provides a practical communication interface for researchers, collaborators, and future validation teams; it is not intended as a clinical decision-support tool in its current form.

### 3.9. Validation-Aware Comparison with Previous Studies

Table 14 compares the proposed CPPN framework with previous EEG treatment-response studies on the same or closely related datasets. This comparison is deliberately validation-aware. Performance values are not direct like-for-like rankings unless the validation unit, subject separation, and model-selection procedure are comparable. Several previous studies reported high accuracies using time-frequency images, transfer learning, recurrent models, ensemble voting, or connectivity-based feature spaces. Where the reporting does not make subject separation explicit, the appropriate interpretation is that high performance supports the reported pipeline under its stated validation setting but should not be equated with nested subject-wise generalisation.

The present comparison supports a cautious but confident methodological conclusion: CPPN provides a compact, transparent, prime-indexed time-domain representation with strong segment-level separability, and the seven-protocol analysis gives a clear pathway for developing the method toward stronger subject-wise evidence. Studies with explicit external cohorts, patient-level validation, or clear subject-wise designs provide useful reference points for the next validation stage. In addition to validation depth, the present framework provides feature-level inspection by mapping the CPPN representation to EEG channel and channel/bin structures, which is not consistently available in deep image-based or transfer-learning EEG pipelines. The distinctive contribution of the present study is the integration of CPPN feature engineering, validation hierarchy, KNN validation-sensitivity testing and interpretable research-dashboard visualisation in one coherent EEG treatment-response research framework. This combination strengthens the originality and rigour of the work because the feature representation, validation structure, and inspection layer are evaluated together rather than reported as disconnected components.

### 3.10. Overall Discussion and Evidential Scope

The strength of this study is its explicit separation of feature-representation performance from validation-depth evidence. This is important for treatment-response modelling because a high segment-level value can be scientifically useful for testing representation quality while still being insufficient for patient-level generalisation. By reporting both high segment-level separability and lower subject-wise estimates, the manuscript presents a more rigorous and transparent contribution than a single best-accuracy report.

The results demonstrate four points. First, CPPN is a strong segment-level representation in the tested EEG cohorts: in the available KNN comparison, CPPN features with fold-internal NCA-selected feature sets achieved higher accuracy than conventional statistical EEG features across all three cohorts, with accuracy gains of 29.53, 16.33, and 11.45 percentage points. In the segment-level 10-fold KNN comparison with training-fold-only normalisation, NCA ranking, and model fitting, CPPN achieved 98.79–99.42% accuracy across the three cohorts. Second, validation design strongly changed the interpretation of performance. When complete subjects were held out, accuracies decreased markedly, showing that unseen-subject evaluation is the principal modelling challenge. The seven-protocol KNN validation hierarchy confirms that validation strictness materially changes the apparent performance of the CPPN representation. Segment-level protocols quantify within-dataset separability and are useful for feature-space benchmarking, but they do not establish external or prospective subject-level generalisation. Subject-wise and nested subject-wise protocols provide more relevant internal estimates for treatment-response modelling, with nested LOSO carrying the greatest evidential weight because feature-count/model selection is separated from final held-out-subject testing. Third, the KNN-only subject-wise validation check shows that the strongest held-out-subject performance occurred in the small Atieh rTMS cohort, whereas the larger rTMS cohort produced more modest subject-wise accuracies. Fourth, nested validation is essential when supervised feature ranking or parameter selection is used, because model selection performed outside the validation loop can produce optimistic estimates. CPPN should therefore be treated primarily as a feature representation whose performance depends on validation design. The proposed framework should be understood as a validation-aware EEG feature-engineering, KNN validation-sensitivity, descriptive inspection, and research-software pipeline. From a complexity perspective, CPPN is a deterministic feature generator that converts each channel into 32 histogram features before optional feature ranking and classification. This is different from CNN pipelines, where convolutional filters are learned and the feature extractor itself becomes part of the trainable model. The available KNN comparison strengthens the feature-representation claim within a segment-level validation boundary, but it does not replace subject-wise LOSO or nested LOSO evidence for held-out-subject evaluation.

The explainability analysis adds methodological value by mapping the selected CPPN representation back to EEG channels and CPPN histogram bins. This links the engineered feature space to interpretable EEG feature groups and enables cohort-specific candidate feature patterns to be described without treating the classifier as a fully opaque model. The channel/bin maps show that selected CPPN features can be summarised as sparse, inspectable candidate structures rather than as an unlabelled high-dimensional vector. The research dashboard extends this interpretability layer by converting abstract CPPN feature vectors into visual panels that combine EEG segment preview, feature-map inspection, and paired cohort-pattern outputs. This software layer makes the model behaviour easier to communicate, supports structured reporting, and provides a practical bridge from algorithmic validation to future prospective software testing.

### 3.11. Limitations and Future Work

The present study also defines a clear next validation pathway and gives a transparent account of the current evidential boundary. First, the segment-level classifier comparison demonstrates strong separability with fold-restricted preprocessing and model fitting, while subject-wise validation remains the more demanding test for future patient-level modelling. Second, held-out-subject performance was cohort- and classifier-dependent, indicating that larger, externally locked cohorts and acquisition-site robustness testing are the next priority. Third, the Atieh data are not publicly downloadable, which limits fully independent reproduction of the complete raw-data pipeline; to support transparency, the processed feature matrices, labels and reproducibility scripts can be shared subject to institutional approvals. Fourth, the prime-indexed CPPN design should be tested in future ablation studies against random and uniformly spaced local sampling, because the present study evaluates the CPPN representation as implemented rather than proving that prime indexing is intrinsically optimal. Fifth, future robustness analysis should include ROC/AUC reporting, calibration analysis, and locked external validation where datasets permit. Finally, the channel/bin feature-pattern analysis is an exploratory interpretability layer that should be assessed with clinician input, test–retest analysis, calibrated decision thresholds, clinical-governance review, risk assessment for incorrect predictions, and prospective workflow studies. These are appropriate next steps for developing the present retrospective EEG feature-engineering framework into externally validated treatment-response research.

## 4. Conclusions

This study developed a validation-aware CPPN-EEG framework for depression treatment-response modelling across SSRI and rTMS cohorts. This work combines a compact prime-indexed time-domain feature representation with available conventional-feature benchmarking, seven-protocol KNN validation, subject-wise validation, nested model-selection control, channel/bin inspection and a research-dashboard interface. Under segment-level 10-fold validation with fold-restricted preprocessing, NCA ranking and model fitting, CPPN + KNN achieved 98.79%, 99.32%, and 99.42% accuracy across the Mumtaz SSRI, small Atieh rTMS, and big Atieh rTMS cohorts, respectively. In the available KNN comparison, CPPN features with fold-internal NCA-selected feature sets improved over conventional statistical EEG features by 29.53, 16.33, and 11.45 percentage points, demonstrating the discriminative value of the CPPN representation within a segment-level validation setting.

The subject-wise and nested analyses add important methodological depth. They show how performance evolves when the validation question moves from segment-level separability to held-out-subject evaluation, with the strongest Tier 2 and Tier 3 KNN results observed in the small Atieh rTMS cohort. This tiered interpretation is scientifically valuable because it prevents a single high accuracy value from dominating the conclusion and instead provides a transparent route for method development. The channel/bin maps and dashboard further support the work by making CPPN feature behaviour inspectable and easier to discuss with research collaborators. Overall, this study presents a validation-aware EEG feature-engineering framework for treatment-response research, supported by processed feature matrices, fold-restricted validation, and an inspectable research-dashboard layer. The next stage is to test the framework on locked external cohorts, expand subject-wise validation with larger samples, and integrate clinician review so that the method can be assessed in future externally validated treatment-response studies.

## Figures and Tables

**Table 1:** Validation depth in EEG-based depression treatment-response studies. Evidential scope summarises the strength of evidence for patient-level generalisation under the reported validation design.

Study	Therapy/Task	Dataset Size	Feature/Model Approach	Main Reported Performance	Main Validation Protocol	No.	Subject Separation Clear?	Evidential Scope	Appropriate Interpretation
[10]	SSRI response	34 participants	WT, STFT and EMD features with logistic regression	91.6% accuracy	Repeated 10-fold cross-validation	1	Partly unclear from reporting	Moderate	Promising early EEG-response prediction; not a validation-hierarchy analysis.
[11]	SSRI response	22 subjects	Nonlinear EEG features, Fisher score and mixture-of-factor analysis	87.4–87.9% accuracy	Leave-n-out randomised permutation cross-validation	1	More explicit than many studies; small sample	Moderate–high	Useful patient-level resampling study, but no systematic comparison of validation strictness.
[12]	Escitalopram outcome	122–222 participants, depending on analysis subset	EEG and clinical features with SVM	82.4% balanced accuracy / accuracy	Cross-validation with additional site-wise generalisability assessment	1–2	Yes for site-wise analysis	High	Stronger generalisation evidence than segment-level CV, but not a segment-to-subject validation hierarchy.
[13]	Antidepressant response	51 individuals	Demographic, EEG and source-space features with ML classifiers	88% accuracy	10-fold cross-validation	1	Participant-level split likely; nesting unclear	Moderate	Supports multimodal EEG prediction, but model-selection and validation depth are not the main focus.
[14]	Antidepressant outcome modelling	518 patients	Pretreatment symptoms and EEG features with gradient-boosted decision trees	C-index up to 0.963	Stratified 5-fold patient-level cross-validation	1	Yes within internal CV	Moderate–high	Large clinical ML study; outcome and validation target differ from segment-level EEG classification.
[15]	Sertraline-predictive EEG signature	309-patient EMBARC trial plus generalisation samples	SELSER resting-EEG signature	Predictive EEG signature; RMSE reported in related comparisons	Cross-validation and cross-study generalisation analyses	1–2	Partly clear; design differs from segment-level ML	Moderate–high	Important EEG-signature work, but not a seven-protocol partitioning comparison.
[16]	SSRI response	30 participants	CWT EEG images with ensemble transfer-learning CNN models	Up to 96.55–99.90% accuracy in reported analyses	10-fold cross-validation on EEG-image/deep-learning pipeline	1	Subject grouping not explicit in accessible reporting	Low–moderate	High image-level performance; unseen-participant interpretation requires clear subject-wise grouping.
[17]	rTMS response	46 participants	CWT-based EEG representations with CRNN and attention	97.1% accuracy	10-fold cross-validation	1	Not clearly subject-wise in accessible reporting	Low–moderate	Strong within-dataset deep-learning performance; not equivalent to nested subject-wise validation.
[18]	Sertraline, bupropion and placebo response	EMBARC subsets	Source-localised effective connectivity with CNN classification	86.8–95.4% accuracy across treatment arms	Validation protocol not fully clear from accessible abstract	1	Not clearly reported from accessible source	Unclear	Modern connectivity/CNN comparator; validation design must be read cautiously before patient-level claims.
[19]	SSRI response	27 patients + 5-patient validation cohort	Relative power, fuzzy entropy, PLI, RFE and ML classifiers	Study-specific classifier results	Internal modelling plus small independent validation cohort	1–2	Yes for external cohort, but sample is very small	High but sample-limited	Stronger validation direction, but external validation remains underpowered.
Present study	SSRI and rTMS response	SSRI: 30; rTMS-small: 15; rTMS-big: 46	MSPCA + CPPN with KNN validation hierarchy and exploratory channel/bin inspection	Seven-protocol KNN validation hierarchy; representative segment-level ACC 98.79–99.42%; best nested LOSO subject ACC 73.33%	Seven validation settings plus KNN-only subject-wise validation checks	7	Yes for subject-wise and nested protocols	Protocol-dependent: segment-level to nested subject-wise evidence	CPPN shows strong fold-wise controlled segment separability, and the subject-wise tiers provide a transparent validation pathway for future externally tested EEG treatment-response modelling.

SELSER: an EEG-based predictive biomarker signature as described in [15]. No.: number of distinct validation protocols reported in each study.

**Table 2:** Dataset, segmentation, and feature-dimensionality summary used in the present analysis.

Dataset/Cohort	Therapy	N	R/NR	Ch.	Rate	Segment	Segments	CPPN/Stat. Features
Mumtaz SSRI	SSRI	30	12/18	20	256 Hz	15 s	579	640/140
Small Atieh Hospital	rTMS	15	9/6	19	500 Hz	15 s	294	608/133
Big Atieh Hospital	rTMS	46	23/23	19	500 Hz	15 s	856	608/133

**Table 3:** Demographic and clinical characteristics of the Mumtaz SSRI dataset.

Characteristic	R	NR
Participants	12	18
Age (years)	40.7±13.0	41.1±12.5
Sex (M/F)	6/6	9/9
Baseline BDI score	18.4±7.4	22.8±12.5
Post-SSRI BDI score	9.1±6.3	22.1±3.3

**Table 4:** Demographic and clinical characteristics of the large Atieh rTMS dataset.

Characteristic	R	NR	*p*-Value
Participants	23	23	–
Age (years)	30.87±12.00	39.00±14.16	0.052
Sex (M/F)	8/15	8/15	0.90
Baseline BDI score	32.5±9.3	28.1±9.4	0.08
Post-rTMS BDI score	8.6±5.9	23.1±8.4	<0.001
Depressive episode length (years)	6.5±8.2	7.9±7.8	0.27

**Table 5:** Validation/data-partitioning protocols used to evaluate the CPPN framework. Segment-level protocols estimate within-dataset EEG segment separability, whereas subject-wise and nested subject-wise protocols provide increasingly conservative internal estimates of generalisation to unseen participants.

Protocol	Validation Tier	Split Unit	Subject Separation	Tuning Isolation	Appropriate Interpretation
Random 80/20 segment split	Tier 1: Segment-level	Segment	No	No	Exploratory segment-level reference; useful for estimating within-dataset separability when segments, rather than subjects, are partitioned.
Segment-level 10-fold cross-validation	Tier 1: Segment-level	Segment	No	No	Within-dataset segment separability; folds are created from segments rather than complete participants.
Nested segment-level cross-validation	Tier 1: Segment-level	Segment	No	Yes	Segment-level separability with fold-internal feature/model selection.
Leave-N-subjects-out (LNSO)	Tier 2: Subject-wise	Subject group	Yes	No	Holds out complete subject groups and trains on the remaining subjects; estimates transfer to unseen participant groups.
Fixed-feature LOSO	Tier 2: Subject-wise	Single subject	Yes	No	Holds out one complete subject at a time. A fixed feature count is pre-specified, and NCA ranking is fitted only on the training subjects.
Nested leave-N-subjects-out	Tier 3: Nested subject-wise	Subject group	Yes	Yes	Holds out a subject group in the outer loop while feature/model selection is performed within the outer-training subjects.
Nested LOSO	Tier 3: Nested subject-wise	Single subject	Yes	Yes	Most conservative internal validation setting; the held-out subject is excluded from normalisation, NCA fitting, feature ranking, feature-count selection, classifier training, and model selection.

**Table 6:** Seven-protocol KNN validation comparison across the three EEG treatment-response cohorts. Tier 1 values summarise segment-level separability, Tier 2 values summarise held-out subject or subject-group performance without nested feature-count selection, and Tier 3 values summarise nested subject-wise validation with feature-count selection separated from final testing. For the random 80/20 split, the reported value is mean accuracy across repeats; for segment-level protocols, the reported value is pooled segment accuracy; for subject-wise protocols, the reported value is subject-majority accuracy.

Tier	Protocol	Split Unit	Subject Sep.	Tuning Isolation	Mumtaz ACC	Small rTMS ACC	Big rTMS ACC	Interpretation
Tier 1: segment-level	Random 80/20 segment split	Segment	No	No	98.36	99.67	99.51	exploratory segment separability
Tier 1: segment-level	Segment-level 10-fold CV	Segment	No	No	98.79	99.32	99.42	controlled segment separability
Tier 1: segment-level	Nested segment-level CV	Segment	No	Yes	98.27	100.00	99.65	segment separability with inner tuning
Tier 2: subject-wise	Leave-N-subjects-out	Subject group	Yes	No	56.67	73.33	52.17	held-out subject-group estimate
Tier 2: subject-wise	Fixed-feature LOSO	Single subject	Yes	No	53.33	80.00	54.35	held-out subject estimate
Tier 3: nested subject-wise	Nested leave-N-subjects-out	Subject group	Yes	Yes	53.33	80.00	54.35	nested subject-group estimate
Tier 3: nested subject-wise	Nested LOSO	Single subject	Yes	Yes	50.00	73.33	47.83	strictest internal estimate

*Note:* Fixed-feature LOSO and nested leave-N-subjects-out produced identical subject-majority accuracy values in these cohorts under the specified feature-count and KNN settings. This reflects the observed outputs of the implemented validation settings and does not by itself imply equivalence of the protocols.

**Table 7:** CPPN + KNN representative comparison of segment-level separability and subject-wise generalisation. Segment-level 10-fold CV used training-fold-only normalisation and model fitting and estimates within-dataset segment separability. Fixed-feature LOSO and nested LOSO estimate held-out-subject performance using subject-majority decisions. Nested LOSO is stricter because feature-count/model selection is confined to training subjects within each outer fold.

Dataset	Seg. ACC (%)	Seg. SEN (%)	Seg. SPE (%)	Fixed-Feature LOSO ACC [95% CI]	Fixed-Feature LOSO SEN [95% CI]	Fixed-Feature LOSO SPE [95% CI]	Nested LOSO ACC [95% CI]	Nested LOSO SEN [95% CI]	Nested LOSO SPE [95% CI]	Interpretation
Mumtaz SSRI	98.79	96.96	100.00	53.33 [36.14–69.77]	25.00 [8.89–53.23]	72.22 [49.13–87.50]	50.00 [33.15–66.85]	25.00 [8.89–53.23]	66.67 [43.75–83.72]	High segment separability (98.79%); modest subject-wise performance after LOSO/nested LOSO (53.33%/50.00%).
Small rTMS	99.32	100.00	98.21	80.00 [54.81–92.95]	100.00 [70.08–100.00]	50.00 [18.76–81.24]	73.33 [48.05–89.10]	100.00 [70.08–100.00]	33.33 [9.68–70.00]	High segment separability (99.32%); strongest small-cohort subject-wise KNN performance (80.00%/73.33%).
Big rTMS	99.42	99.31	99.52	54.35 [40.18–67.85]	39.13 [22.16–59.21]	69.57 [49.13–84.40]	47.83 [34.12–61.86]	34.78 [18.81–55.11]	60.87 [40.79–77.84]	High segment separability (99.42%); modest larger-cohort subject-wise performance (54.35%/47.83%).

**Table 8:** KNN-only subject-wise validation comparison for the Mumtaz SSRI cohort. Subject-level predictions were obtained by majority voting across the held-out subject’s segment predictions. Non-KNN classifier rows are not included because this subject-wise validation check is reported as a KNN-only analysis.

Protocol	Classifier	Subject ACC [95% CI]	Subject SEN [95% CI]	Subject SPE [95% CI]	TP/FN	TN/FP	Parameters
Tier 2 fixed-feature LOSO	KNN	53.33 [36.14–69.77]	25.00 [8.89–53.23]	72.22 [49.13–87.50]	3/9	13/5	K = 5; Euclidean; fixed features = 243
Tier 3 nested LOSO	KNN	50.00 [33.15–66.85]	25.00 [8.89–53.23]	66.67 [43.75–83.72]	3/9	12/6	K = 5; Euclidean; nested features median = 200, mode = 100

**Table 9:** KNN-only subject-wise validation comparison for the Small rTMS cohort. Subject-level predictions were obtained by majority voting across the held-out subject’s segment predictions. Non-KNN classifier rows are not included because this subject-wise validation check is reported as a KNN-only analysis.

Protocol	Classifier	Subject ACC [95% CI]	Subject SEN [95% CI]	Subject SPE [95% CI]	TP/FN	TN/FP	Parameters
Tier 2 fixed-feature LOSO	KNN	80.00 [54.81–92.95]	100.00 [70.08–100.00]	50.00 [18.76–81.24]	9/0	3/3	K = 5; Euclidean; fixed features = 587
Tier 3 nested LOSO	KNN	73.33 [48.05–89.10]	100.00 [70.08–100.00]	33.33 [9.68–70.00]	9/0	2/4	K = 5; Euclidean; nested features median = 300, mode = 587

**Table 10:** KNN-only subject-wise validation comparison for the Big rTMS cohort. Subject-level predictions were obtained by majority voting across the held-out subject’s segment predictions. Non-KNN classifier rows are not included because this subject-wise validation check is reported as a KNN-only analysis.

Protocol	Classifier	Subject ACC [95% CI]	Subject SEN [95% CI]	Subject SPE [95% CI]	TP/FN	TN/FP	Parameters
Tier 2 fixed-feature LOSO	KNN	54.35 [40.18-67.85]	39.13 [22.16–59.21]	69.57 [49.13–84.40]	9/14	16/7	K = 5; Euclidean; fixed features = 377
Tier 3 nested LOSO	KNN	47.83 [34.12–61.86]	34.78 [18.81–55.11]	60.87 [40.79–77.84]	8/15	14/9	K = 5; Euclidean; nested features median = 377, mode = 587

**Table 11:** Available KNN segment-level comparison between conventional statistical EEG features and CPPN features. Statistical-feature values are compared with CPPN values from segment-level 10-fold CV KNN results, where the full CPPN representation is the source feature matrix and NCA ranking is fitted inside the training folds before selecting the stated feature count. The comparison is therefore matched at the classifier and segment-level validation level, but the CPPN side is an NCA-selected CPPN + KNN result rather than an unselected full-feature CPPN result.

Dataset	Statistical Feature Matrix/Dimension	Stat. ACC	Stat. SEN	Stat. SPE	CPPN Full Dim.	Selected CPPN Features	NCA Scope	CPPN ACC	CPPN SEN/SPE	ACC Gain
Mumtaz SSRI	579×140	69.26	50.00	81.95	640	243	training-fold only	98.79	96.96/100.00	+29.53 pp
Small Atieh rTMS	294×133	82.99	94.51	64.29	608	587	training-fold only	99.32	100.00/98.21	+16.33 pp
Big Atieh rTMS	856×133	87.97	93.59	82.10	608	377	training-fold only	99.42	99.31/99.52	+11.45 pp

*Note:* pp = percentage points. The CPPN full dimension is the source feature-vector size before fold-internal NCA ranking. The reported CPPN KNN values use the selected CPPN feature counts shown in the table, with NCA ranking fitted only on training folds. The comparison is matched at the classifier and segment-level validation level, but it is not an unselected full-feature CPPN comparison. Held-out-subject performance is evaluated separately using fixed-feature LOSO and nested LOSO protocols.

**Table 12:** Segment-level 10-fold CV compared with nested segment-level CV using CPPN features with KNN.

Dataset	10-Fold ACC	Nested ACC	Δ ACC	10-Fold SEN	Nested SEN	10-Fold SPE	Nested SPE	Nested Feat. Med./Mode
Mumtaz SSRI	98.79	98.27	−0.52	96.96	96.52	100.00	99.43	200.00/100.00
Small rTMS	99.32	100.00	0.68	100.00	100.00	98.21	100.00	95.00/56.00
Big rTMS	99.42	99.65	0.23	99.31	99.54	99.52	99.76	377.00/300.00

ACC, SEN, and SPE denote accuracy, sensitivity, and specificity, respectively. Δ ACC denotes nested segment-level CV minus segment-level 10-fold CV.

**Table 13:** Candidate feature-pattern interpretability summary derived from CPPN channel/bin mapping. Exploratory top channel is derived from the curated candidate-marker analysis. No FDR-significant subject-level markers were identified in any cohort; all findings are hypothesis-generating.

Dataset	Therapy	Top Channel (LOSO)	Subject-Level Feature-Pattern-Score Trend	Interpretability Value
Mumtaz	SSRI	Pz	Exploratory responder/non-responder trend with overlap; no FDR significance	Parietal channel/bin candidate pattern; external SSRI-response validation required
Small Atieh Hospital	rTMS-small	Fp2	Exploratory trend in small cohort (n=15); no FDR significance	Preliminary frontal channel/bin candidate pattern; larger rTMS validation required
Big Atieh Hospital	rTMS-big	Cz	Exploratory trend; no FDR significance	Central channel/bin candidate pattern; prospective rTMS validation required

**Table 14:** Validation-aware comparison between the proposed framework and existing EEG treatment-response approaches.

Study	Task	Dataset	Feature/Model Approach	Main Reported Performance	Validation Protocol	Subject Sep.	Validation-Aware Interpretation
[10]	SSRI	Mumtaz	WT, STFT, EMD and logistic regression	ACC 91.60; SEN 90.00; SPE 90.00	Repeated 10-fold CV	Partly unclear	Useful early comparator; validation depth is not equivalent to the present seven-protocol hierarchy.
[16]	SSRI	Mumtaz	CWT images and ensemble transfer learning	ACC up to 99.90% across reported analyses; representative ACC 96.55; SEN 96.01; SPE 96.95	10-fold CV on EEG-image pipeline	Not explicit	High image-level performance; not directly comparable to nested LOSO subject-majority results.
[34]	SSRI	Mumtaz	Three transfer-learning models with BLSTM	ACC 98.84; SEN 97.80; SPE 99.60	Deep-learning CV; subject grouping unclear from reported summary	Unclear	Strong reported accuracy, but validation unit must be considered before subject-level claims.
[35]	SSRI	Mumtaz	Effective-connectivity features with hybrid convolutional/recurrent deep learning	ACC 98.33	Source-reported validation; subject grouping requires source-level checking	Unclear	High reported accuracy; not a direct ranking against nested subject-wise validation.
[36]	rTMS	Small Atieh	Effective-connectivity images with an ensemble of pre-trained CNN models	Source-reported performance; exact split details require source verification	Deep-learning validation; subject separation unclear from accessible summary	Unclear	Comparator uses a heavier image/connectivity model; validation context differs from the present hierarchy.
[37]	rTMS	Big Atieh	Attention-based convolutional recurrent deep neural networks	Source-reported performance; exact split details require source verification	Deep-learning validation; subject separation unclear from accessible summary	Unclear	High reported performance cannot be directly compared with nested LOSO without matching validation design.
[17]	rTMS	Big Atieh	Convolutional recurrent neural network with attention	ACC 97.10; SEN 97.30; SPE 97.00	10-fold CV	Not clearly subject-wise	Strong within-dataset deep-learning result; validation design differs from nested subject-wise testing.
[38]	rTMS	Big Atieh	Hybrid convolutional recurrent networks using raw EEG	ACC 98.51; SEN 98.64; SPE 98.36	Not clearly subject-wise in accessible reporting	Unclear	High reported accuracy with a complex deep-learning pipeline; comparison should be protocol-aware.
Present study	SSRI	Mumtaz SSRI	MSPCA + CPPN with seven-protocol KNN validation hierarchy	KNN segment ACC 98.79; nested LOSO subject ACC 50.00	Seven-protocol hierarchy plus KNN-only subject-wise validation check	Yes for subject-wise tiers	Strong segment-level separability; subject-wise generalisation remains limited and validation-dependent.
Present study	rTMS	Small Atieh rTMS	MSPCA + CPPN with seven-protocol KNN validation hierarchy	KNN segment ACC 99.32; nested LOSO subject ACC 73.33	Seven-protocol hierarchy plus KNN-only subject-wise validation check	Yes for subject-wise tiers	Strong segment-level separability; strict subject-wise performance is cohort-dependent.
Present study	rTMS	Big Atieh rTMS	MSPCA + CPPN with seven-protocol KNN validation hierarchy	KNN segment ACC 99.42; nested LOSO subject ACC 47.83	Seven-protocol hierarchy plus KNN-only subject-wise validation check	Yes for subject-wise tiers	Validation hierarchy reveals a large segment-to-subject generalisation gap.

Validation protocols differ across studies; therefore, performance values should be interpreted according to validation design rather than as direct like-for-like rankings. Abbreviations: BLSTM: Bidirectional Long Short-Term Memory; DE: Differential Evolution. SEN and SPE values for [10] are as reported in the original study.

## Data Availability

The Mumtaz SSRI dataset analysed in this study is publicly available from the source described in the original dataset publication [10,24] and through Figshare at https://doi.org/10.6084/m9.figshare.4244171.v2. The Atieh Hospital rTMS datasets are not publicly available because they contain clinical participant data and are subject to institutional and ethical restrictions. Requests for access to the private rTMS data and derived analysis materials may be directed to the corresponding author and will be considered subject to internal approval, institutional data-governance requirements, and participant-confidentiality restrictions where appropriate. Derived CPPN feature matrices, class labels, and analysis scripts may be made available by the corresponding author upon reasonable request, subject to institutional approvals and applicable data-governance restrictions.
